# Operational Global Actual Evapotranspiration: Development, Evaluation, and Dissemination

**DOI:** 10.3390/s20071915

**Published:** 2020-03-30

**Authors:** Gabriel B. Senay, Stefanie Kagone, Naga M. Velpuri

**Affiliations:** 1 U.S. Geological Survey (USGS), Earth Resources Observation and Science (EROS) Center, North Central Climate Adaptation Science Center, Boulder, CO 80303, USA; 2 ASRC Federal Data Solutions LLC, Contractor to the U.S. Geological Survey EROS Center, Sioux Falls, SD 57198, USA; skagone@contractor.usgs.gov (S.K.); N.Velpuri@cgiar.org (N.M.V.); 3 International Water Management Institute—Colombo, 127, Sunil Mawatha, Battaramulla 10120, Colombo, Sri Lanka

**Keywords:** actual evapotranspiration, global, SSEBop model, MODIS, remote sensing, drought monitoring

## Abstract

Satellite-based actual evapotranspiration (ETa) is becoming increasingly reliable and available for various water management and agricultural applications from water budget studies to crop performance monitoring. The Operational Simplified Surface Energy Balance (SSEBop) model is currently used by the US Geological Survey (USGS) Famine Early Warning System Network (FEWS NET) to routinely produce and post multitemporal ETa and ETa anomalies online for drought monitoring and early warning purposes. Implementation of the global SSEBop using the Aqua satellite’s Moderate Resolution Imaging Spectroradiometer (MODIS) land surface temperature and global gridded weather datasets is presented. Evaluation of the SSEBop ETa data using 12 eddy covariance (EC) flux tower sites over six continents indicated reasonable performance in capturing seasonality with a correlation coefficient up to 0.87. However, the modeled ETa seemed to show regional biases whose natures and magnitudes require a comprehensive investigation using complete water budgets and more quality-controlled EC station datasets. While the absolute magnitude of SSEBop ETa would require a one-time bias correction for use in water budget studies to address local or regional conditions, the ETa anomalies can be used without further modifications for drought monitoring. All ETa products are freely available for download from the USGS FEWS NET website.

## 1. Introduction

The estimation of actual evapotranspiration (ETa) is an important activity in crop and water management. As a key water budget component in the hydrologic cycle, ETa is responsible for the exchange of mass and energy between land surfaces and atmosphere, thereby ensuring the continuity of the hydrologic cycle and recycling 60–75% of total terrestrial precipitation [1,2]. ETa is composed of two sub-processes—evaporation from surface and transpiration through plants—which are difficult to measure directly, especially over large areas. Hence, estimation and mapping at a large scale is restricted to indirect methods. The two most common principles in estimating ETa are water balance and energy balance approaches [3]. In water balance approaches, ETa is estimated as a function of soil moisture by tracking precipitation and other contributions (e.g., irrigation) in a controlled volume (e.g., root-zone storage). This is the physics-based simulation of ETa as implemented by prognostic hydrologic models, commonly described as land surface models (LSM) such as the Global Land Evaporation Amsterdam Model (GLEAM) [4] and the Variable Infiltration Capacity (VIC) model [5]. ETa can also be estimated diagnostically using energy balance approaches as the residual of the surface energy balance components where the difference between available net radiation and outgoing (sensible plus ground heat) flux is attributed to latent heat (LE) flux. LE can be converted into ETa using the latent heat of a vaporization constant (e.g., SEBAL [6]; ALEXI [7]; METRIC [8]). In all methods, ETa is generally estimated as a fraction of the potential evapotranspiration (PET) or net radiation, representing maximum evapotranspiration (ETa). The main difference between water balance and energy balance approaches is in the calculation of the ET fraction (ETf), which is a function of soil moisture for the water balance approach, but calculated from remotely sensed land surface temperature (LST) in cases of the energy balance approach. Furthermore, ET fractions can be calculated from satellite-derived vegetation indices (VI) such the Normalized Difference Vegetation Index (NDVI). VI-based ET fractions can be used to estimate ETa directly [9,10,11,12] or in combination with water balance approaches [13,14]. Other sources of ETa datasets are generated from the upscaling and regionalizing of station-based flux tower eddy covariance measurements in combination with remote-sensing-based environmental parameters such as the gridded flux net by the Max Planck Institute [15,16]. 

The quantification of ETa is prone to large uncertainties due to its dependence on the quality of model inputs in addition to weaknesses in model structure and parameter accuracy. Mueller et al. [17] attributed uncertainties in global ETa to the lack of reference observations. However, the desired level of accuracy for ETa depends on its application and can be grouped into two categories: (1) water budget and (2) drought monitoring. While absolute accuracy is critical for water budget applications, the year-to-year consistency is more useful for drought monitoring purposes. There exist several global ETa datasets from both land surface (prognostic) and remote sensing (diagnostic) approaches, but only two are presently available for free download and use by the user community on an operational basis. These publicly available sources are: (1) MOD16 [10] (https://lpdaac.usgs.gov/products/mod16a2v006/, last accessed 1/29/2020) and (2) the Operational Simplified Surface Energy Balance Model (SSEBop) [18]. MOD16 ET data have been published and extensively referenced by various researchers [19,20]. On the other hand, despite its online availability for various applications, the global SSEBop ETa product has not been supported by a publication. The main objectives of this study are: (1) to present the methods used in creating the global SSEBop ETa product, (2) to conduct a first-order evaluation using flux tower eddy covariance ETa and precipitation data, and (3) to introduce the SSEBop ETa anomaly as a drought monitoring tool.

## 2. Materials and Methods

### 2.1. Data

The global land surface is diverse, consisting of mountains and valleys with land cover ranging from barren and hot deserts to thick and temperate forests. These diverse land surface characteristics are captured by spatially explicit geospatial datasets that are inputs to the SSEBop modeling approach as summarized in Table 1.

Land surface temperature (LST, also represented by Ts as a variable) is one of the two important drivers in the model along with reference evapotranspiration (ETo). We used dekadal (10-day) LST data provided by the US Geological Survey (USGS) Earth Resources Observation and Science (EROS) Center (Moderate Resolution Imaging Spectroradiometer (MODIS) LST, https://doi.org/10.5066/P9BT8RIP, last accessed 1/29/2020). The LST dataset spatial resolution is 1 km with a global extent of 80° North to 60° South and 180° West to 180° East, starting in 2003 and ranging up to the current year (2020). Emissivity data are provided along with the LST dataset using the same raster and temporal properties. The daily maximum air temperature (Ta) dataset is key for determining the cold/wet boundary limit parameter (Tc). To utilize the best available datasets globally, we spatially merged Ta from Daymet (https://daymet.ornl.gov/, last accessed 1/29/2020) [21] for North America (average for 1980–2010) and Ta from WorldClim (average for 1970–2000) (http://www.worldclim.org, last accessed 1/29/2020) [22] for the remaining parts of the globe into one comprehensive dataset. We used a climatology dataset for ETo (2003–2012) from the Global Data Assimilation System (GDAS) provided by the USGS Famine Early Warning Systems Network (FEWS NET) Data Portal (https://earlywarning.usgs.gov/fews/product/81, last accessed 1/29/2020) and climatological potential PET provided by the International Water Management Institute (IWMI) at a 10 km resolution. The values from GDAS (daily at a 100 km spatial resolution) were downscaled to 10 km using spatial patterns and statistics derived from the higher resolution IWMI PET dataset [23]. This allowed the creation of improved spatial (10 km) and temporal (daily) resolutions by combining a coarse (100 km) daily GDAS with a finer (10 km) monthly climatology IWMI PET.

The Normalized Difference Vegetation Index (NDVI, MYD13A2.006, 1 km, 2003 to 2020), emissivity and albedo surface reflectance (MCD43A3.006 MODIS Albedo Daily 500 m, 2000 to 2020) were used for model parameterization and adjustments as follows. The NDVI was used to locate cold/wet pixels that are used to calibrate the correction coefficient (c factor), which converts Ta into the cold/wet reference limit (Tc). Emissivity and albedo data were used to adjust the relatively low LSTs (Ts) observed over unexpectedly high emissivity (>0.965) and albedo (>0.25) pixels in sparsely vegetated (NDVI < 0.25) surfaces. High albedo (>0.25) and emissivity (>0.965) LSTs were adjusted to warmer Ts to avoid a false sense of cooler temperatures that would be mistaken for high ETa areas when they occur over sparse (NDVI < 0.25) vegetation landscapes. These adjustments tend be needed in arid and semi-arid areas where the overall ET is very low to minimize artifacts that appear in such low ET surfaces. 

### 2.2. SSEBop Model Approach

Since the publication of the SSEBop model in 2013 by Senay et al. [18], the model has been applied over diverse hydroclimatic regions with modifications made to model setup and parameter thresholds, depending on the availability of input datasets. However, the overall principle has remained the same, in that SSEBop is a two-parameter model with: (1) the temperature difference (dT) and (2) a cold/wet reference limit (Tc).

**Figure 1:**
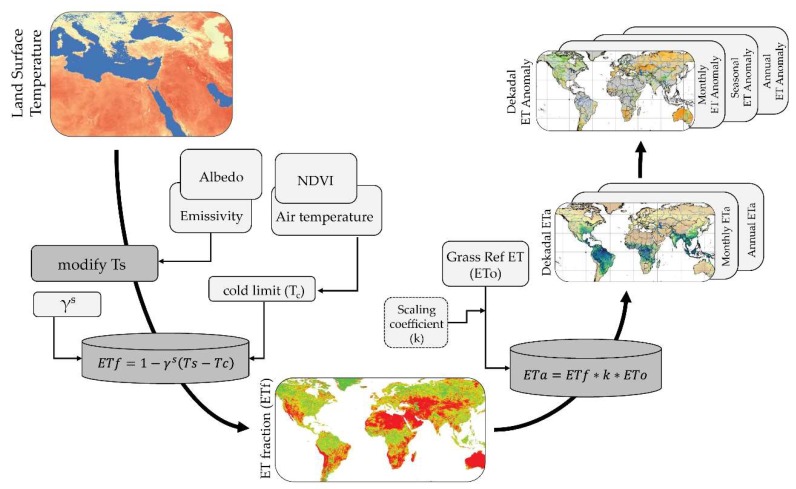
Overview of the SSEBop modeling process, adapted from Senay et al. [24], from the input data to the final products posted to the United States Geological Survey (USGS) Famine Early Warning Systems Network (FEWS NET) Data Portal (https://earlywarning.usgs.gov/fews/).

Figure 1 shows a general flow diagram of the implementation of the SSEBop. Important forcing inputs (Ts and ETo) and model parameters (air temperature, NDVI, γ^s^, albedo, and emissivity) are shown. Final products are posted to the FEWS NET website (https://earlywarning.usgs.gov/fews/). LST, as noted in Section 2.1, is the most important model forcing variable. The estimation of ETa with SSEBop is a two-step process where the ET fraction is first calculated using Ts, then ETa is calculated as the product of ETf and the maximum ET derived from ETo. The ETf is calculated on a per-pixel basis using Equation (1):

(1) $$\mathrm{ETf}=1-\gamma^{s}\left(\mathrm{Ts}-\mathrm{Tc}\right)$$

where *ETf* is the dekadal ET fraction for each pixel nominally ranging between 0 and 1, and γ^s^ is the ‘surface’ psychrometric constant over a dry-bare surface (it is the same as the inverse of the dT parameter in Senay et al. [18]). Ts (K) is derived from MODIS LST, and Tc is the coldest/wettest surface temperature (K) limit, which is derived from *Ta* [18]. The constant 1 represents the ET fraction value during maximum ET (i.e., when Ts = Tc). ETa is then determined on a per-pixel basis using Equation (2):

(2) ETa=ETf∗k∗ETo

where ETa is the actual ET (mm), and ETo is the (grass-reference) potential ET (mm), which refers to water that is transpired by grass, completely shaded, uniform in height, and never short of water; k is a scaling coefficient of 1.25, which scales-up the grass-reference ETo to an alfalfa reference type for maximum ETa, since alfalfa is an aerodynamic rougher crop than grass and therefore preferred to represent the natural environment modeled.

As described in Senay et al. [18], the dT parameter was one of the key developments in simplifying the ET computation process by completely eliminating the need to manually determine the hot reference pixel. This allowed the model to be operationalized for use by the US Department of the Interior’s WaterSMART (Water Sustain and Manage America’s Resources for Tomorrow) Program and by FEWS NET projects. Senay [25] re-formulated the SSEBop model using the principle of satellite psychrometry where the wet- and dry-bulb parameters are associated with the *Tc* and *Ts*, respectively. 

The surface psychrometric constant (γ^S^) is defined as

(3) $$\gamma^{S}=\frac{C_{p}\rho }{R_{n}*r_{\mathrm{ah}}}$$

where C_p_ is defined as the specific heat of air at constant pressure 1.013 * 10^−3^ (MJ/(kg.°C)), ρ is the density of air (kg/m^3^), R_n_ is the daily average net radiation (MJ/(m^2^.day)) [25], and r_ah_ is the aerodynamic resistance over bare soil, defined as 110 s/m [25].

The second important model parameter is the cold/wet reference limit (*Tc*), which is determined from the daily maximum air temperature using the c factor (Table 2).

(4) Tc=c∗Ta

where Tc (K) is the cold/wet boundary limit or wet bulb, Ta is the maximum air temperature (K) for the period, and c is the correction coefficient, which is developed as the ratio of Ts and Ta at well- vegetated pixels. 

The cold/wet boundary (wet bulb) is used to define the ET rate at a well-watered and well-vegetated surface based on the assumption that there is little or no sensible heat transfer because the air and surface temperatures are identical at this surface and thus produce ET at the potential rate. The c factor is based on the ratio (Tcorr) of Ts to Ta on pixels that meet the condition of NDVI >= 0.7 (well-vegetated pixel). Furthermore, the temperature difference between Ta and Ts is restricted to between −10 and 5 K (to avoid extreme calibration points when using the Aqua LST and this particular Ta dataset) and *Ts* has to be > 270 K to exclude cloudy pixels (Table 2). The Tcorr (i.e., Ts/Ta) values that meet all three conditions are then summarized for each MODIS sub-tile. MODIS sub-tiles are generated by sub-diving each MODIS tile into 25 sub-tiles (5 × 5 tiles). The sub-tiling improves the optimization of the c factor that is more appropriate for each sub-tile, thereby improving the spatial accuracy of the model. The final c factor is then determined as the 5th percentile (mean minus two standard deviations) from the distribution of Tcorr values for each sub-tile:

(5) $$c={\mathrm{Tcorr}}_{\mathrm{mean}}-2*\mathrm{STD}$$

where Tcorr_mean_ is the mean Tcorr value for each sub-tile and STD is the standard deviation. The Tcorr value is reduced by two times the STD to eliminate outliers and arrive at the coldest/wettest pixel values at the 5th percentile. 

The c factor is generated only when a sub-tile has more than 30 pixels that meet the criteria. If a c value cannot be determined, the c value of neighboring tiles is used. This approach is reasonable because of the relatively small size of the tile and because hydroclimatic conditions tend to change gradually. When neighboring sub-tiles do not have valid c factors, median c factors are used to create a c factor raster that covers the entire study area. 

Once c factors are generated the major challenge for generating ET fractions is cloud-related issues (clouds, cloud shadows, and contamination). Thus, a missing ETf is filled using the BABA (before-after-before-after) algorithm to estimate the ETa for a given dekad “i”. An ETf is considered invalid if it is greater than 1.3; however, ETf values between 1.05 and 1.3 are capped at 1.05 [25]. Using the BABA algorithm, if the ETf value is greater than 1.3, then the ETf from the previous (before) dekad (i − 1) is used. If the “before” dekad is invalid, then the dekad after (i + 1) is considered. If that fails, then we go back to the two previous dekads (i − 2), or forward to the two subsequent dekads (i + 2), in that order. After four attempts to fill the missing data using adjacent periods have failed (when BABA fails) due to longer periods of cloud (e.g., during monsoon seasons in India or the Amazon), the median ETf value (2003–2017) for the same period is considered as a reasonable ETf estimate. 

In order to document whether a pixel is observed (calculated) or filled with a certain approximation, a numerical quality assurance (QA) band is created. The QA raster contains values from 1 to 6, where “1” represents the availability of a current (i) dekad ETf value and no need for filling; “2”: missing ETf is filled with the previous dekad (i − 1) ETf; “3”: missing ETf is filled with (i + 1); “4”: missing ETf is filled with (i − 2); “5”: missing ETf is filled with (i + 2); and “6”: missing ETf is filled with the median ETf value.

Once the ETa is calculated for each dekad, two more modifications were made to the ETa over desert areas and waterbodies. First, a desert area was defined when the maximum NDVI value (for 2003–2017) was less than 0.2. For desert areas such as The Sahara, ETa was capped at 32% of the calculated value. The value was determined based on a comparison with rainfall data where ETa was assumed to be equal to rainfall in such dry environments. Climate Hazards Group InfraRed Precipitation with Station (CHIRPS) data were used for the rainfall [27]. Secondly, ETa over known waterbodies was assigned 85% of the potential ET (R. Allen, Kimberly Research and Extension Center University of Idaho, verbal communication, 2013). The waterbodies were defined using the MODIS Land Cover product (MCD12Q1), where we extracted water (ID = 0) from the raster file, resampled it to a 1 km spatial resolution (from 500 m), then extracted the inland water features. To avoid the inclusion of seasonally vegetated pixels near waterbodies, a water occurrence raster was used to define waterbodies, i.e., a pixel was declared water if the land cover map identifies the pixel as water (ID = 0) in all years (2003–2013). 

Temporal aggregation was achieved by multiplying the dekadal ETf and dekad-total ETo. A dekadal global ETa was created for all dekads over 16 years (2003–2018). The dekadal ETa data were further aggregated to generate monthly, seasonal, and annual ETa grids. Graphics for all three temporal scales (dekad, month, and year) were created for visual interpretation of the data and posted to the FEWS NET website (https://earlywarning.usgs.gov/fews/). Supporting data and graphics are available as a USGS Data Release as indicated in the Supplementary Materials section.

### 2.3. Evaluation of SSEBop ETa Estimates Using Eddy Covariance Flux Towers

To evaluate the ETa dataset, monthly modeled ETa estimates were compared with monthly ETa eddy covariance flux tower data (aggregated from daily) provided by FluxNet 2015 (https://fluxnet.fluxdata.org/, last accessed 1/29/2020). FluxNet 2015 is a curated dataset where the regional flux networks in North, Central and South America, Europe, Asia, Africa, and Australia contribute data to create a cohesive global network of eddy covariance measurements. The daily data were converted to ETa (mm/day) and aggregated to monthly using the proportionality constant between energy and ET depth in mm as:

(6) $$\mathrm{ETa}=\frac{\mathrm{LE}}{\lambda }$$

where λ is the latent heat of vaporization (2.45 MJ/kg) and latent heat (LE) is in comparable energy units of MJ/(m^2^.day) and ETa in mm/day (i.e., 1 MJ/(m^2^.day) = 0.408 mm/day with a water density of 1000 kg/m^3^) [14]. There are 165 Tier 1 flux tower sites in the FluxNET 2015 database. In order to show globally distributed sample comparisons, we arbitrarily selected two sites per continent (different land cover types where available), for a total of 12 sites with valid data since 2003, a starting period for SSEBop ETa. We also limited the locations to those that had at least three years of flux tower measurements (the Brazil site had only two years of data, with only two sites available in South America). The selected sites included: Australia: AU-Wom, AU-DaP; North America: CA-SF1, US-Ne1; South America: BR-Sa3, AR-SLu; Asia: CN-Cng, CN-Du2; Europe: DE-Obe, DE-She; and Africa: ZM-Mon, ZA-Kru. One site for each continent was over forest land cover and the second was on grassland or savanna. The modeled monthly ETa was compared to the observed latent heat flux-based ETa. Standard statistical metrics such as correlation coefficient (r), root mean square error (RMSE), normalized RMSE (percent derived from the range or mean), and percent bias (from the mean) were generated. The normalized RMSE using the range was used because of the seasonality of ETa, creating a large range in ETa values. The mean was used to determine the percent bias. 

Energy balance closure (EBC) error has always been an issue with eddy covariance (EC) tower datasets [28,29]. We calculated the EBC (ratio of the sum of latent and sensible fluxes to net radiation minus ground heat flux) on a daily time step for each of the 12 sites. We used thresholds of EBC = 0.7 and 1.0 to identify potential under-estimation with low closure (EBC < 0.7) and over-estimation with high closure (EBC > 1.0). The energy balance residual correction was computed using the corrected variables for latent heat (LE_CORR) and sensible heat (H_CORR) in addition to net radiation (NETRAD) and ground heat (G_F_MDS) for the data provided. The percentage of days with low and high EBC are reported to indicate the reliability of the eddy covariance data for interpreting the accuracy metrics against SSEBop ETa.

### 2.4. Evaluation of SSEBop ETa Using Annual Water Budget at Pixel and Basin Scales

Considering the limited coverage of flux towers, we also conducted a pixel-based and basin-scale annual water budget analysis. The major assumption was that the storage change for each spatial scale was negligible at an annual time scale. Therefore, we created mean annual precipitation (PPT) totals (2006–2015) from the Multi-Source Weighted-Ensemble Precipitation (MSWEP) data [30] and mean annual ETa from SSEBop data for the same time period. The pixel-based analysis showed the annual difference between PPT and ETa as a map with positive values showing runoff generation pixels, while negative values indicating sink areas such as wetlands and large irrigated deltas where there was more ETa than direct rainfall over the pixel. We selected one major river basin for each continent and spatially averaged the PPT and ETa data. The ETa/PPT coefficient (ET_coeff_) for the six major river basins over 10 years (2006–2015) was calculated as:

(7) $${\mathrm{ET}}_{\mathrm{coeff}}=\frac{\mathrm{ETa}}{\mathrm{PPT}}$$

where ET_coeff_ is the fraction of basin-average ETa in relation to basin-average PPT for each year. ET_coeff_ provides a sense of the relative amount of precipitation that is partitioned between ETa and runoff, with tropical desert areas having a high ET_coeff_ compared to wet temperate regions [31].

### 2.5. ETa Anomalies for Drought Monitoring

One of the main uses of the global ETa datasets is drought monitoring, which relies on relative changes from a normal climate. The ETa anomalies are computed as the percentage of monthly, seasonal and annual median values for the corresponding aggregation periods. The median is calculated from the available historical data (2003–2017) constituting a 15-year normal. Unlike applications in water resources and hydrological water budget studies, the absolute accuracy of ETa is not critical for drought monitoring. However, a consistent global ETa dataset that captures the spatiotemporal variability of landscape stress from lack of moisture allows the monitoring/early warning of droughts at a relatively high spatial resolution (1 km). Global and regional ETa anomaly products are regularly posted to the FEWS NET website (https://earlywarning.usgs.gov/fews/).

## 3. Results and Discussion

### 3.1. SSEBop ETa Estimates

**Figure 2:**
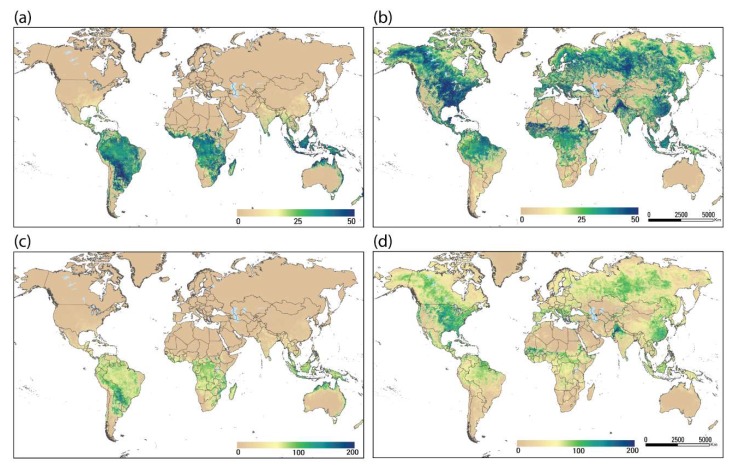
(**a**) Actual ET (mm) for dekad 1, January 2018 (1.1.2018–10.1.2018), (**b**) actual ET (mm) for dekad 1, July 2018 (1.7.2018–10.7.2018), (**c**) actual ET (mm) for the month of January 2018 (1.1.2018–31.1.2018), and (**d**) actual ET (mm) for the month of July 2018 (1.7.2018–31.7.2018).

Model outputs were created for every dekad. A typical product is illustrated in Figure 2a,b for the first dekad of January and July 2018, respectively. One can see the active growing seasons in January for the southern hemisphere and in July for the northern hemisphere, with ETa values in the range of 20–60 mm per dekad. Monthly totals were created by aggregating the three dekads in each month; for example, January and July 2018 SSEBop ETa values are illustrated in Figure 2c,d, respectively. In the month of January, South America and southern Africa showed high ETa values due to an active growing season. A typical range for healthy vegetation/crops is about 150–200 mm per month during a peak season, represented by dark blue-green colors. In July, the active growing seasons are visible in the northern hemisphere, such as in the United States (>150 mm) and the Indus Valley (South Asia) (>200 mm). Overall, the spatial distribution of the ETa pattern for each time period corresponded well with the vegetation cover and rainfall/irrigation patterns of the globe, highlighting where and how much ETa was generated for a given time period of the year.

### 3.2. Evaluation of ETa Estimates Using Eddy Covariance (EC) Data

**Figure 3:**
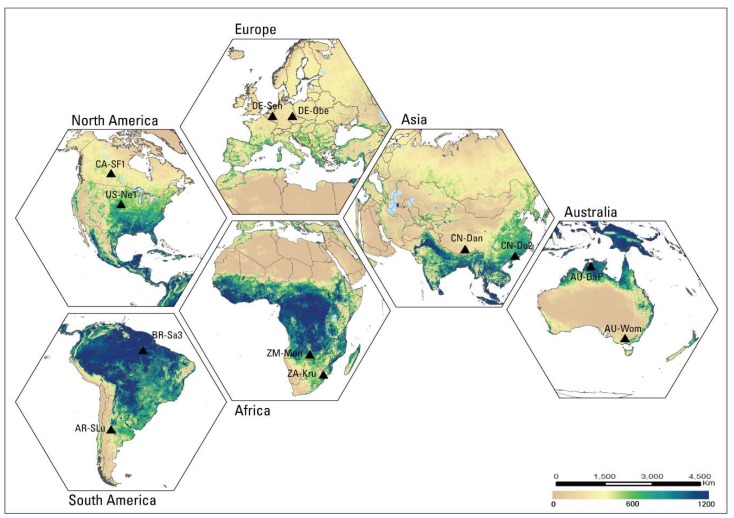
2018 annual ETa map (mm) with 12 flux tower locations. There are two sites on each of the six continents.

Twelve EC flux tower sites from around the world were used to evaluate monthly SSEBop ETa. The locations of the 12 selected sites are shown in Figure 3, with the annual ET estimates for 2018 as the base map. The time period for the EC data was 2003–2014, based on the availability of flux tower measurements.

Overall, the two datasets compared favorably with a correlation coefficient r as high as 0.87 with some exceptions (as low as r = 0.09) (Table 3). The strongest correlations occurred in Australia at the AU-Wom site (Evergreen Broadleaf Forests, r = 0.87, RMSE = 13.6 mm) and Europe at the DE-Obe site (Evergreen Needleleaf Forests, r = 0.82, RMSE = 15.8 mm). The lowest correlations were with the sites in South America: one in Argentina, AR-SLu (Mixed Forest, r = 0.09, RMSE = 43.7.1 mm) and one in Brazil, Br-Sa3 (Evergreen Broadleaf Forests, r = 0.32, RMSE = 7.5 mm). Although ETa seasonality seemed to be captured well by the SSEBop model, as demonstrated by a relatively strong r, large biases ranging from 3 to 50% were observed in both negative and positive directions. While the model can be subject to biases as a result of various factors from model structure to input sources, the EC flux tower ETa was also subject to potential biases and uncertainties, as indicated by the relatively large energy balance closure (EBC) error percentages. For example, the DE-Obe station in Europe showed 34.2% of the daily observed data with an EBC less than 0.7 and 38.3% with more than 1.0 (Table 4). Similarly, most of stations had a high percentage of days with an EBC outside the desired range. Interestingly, these datasets are supposed to be corrected for EBC error, but still showed a high percentage of the days away from the desired value of 1.0.

**Figure 4:**
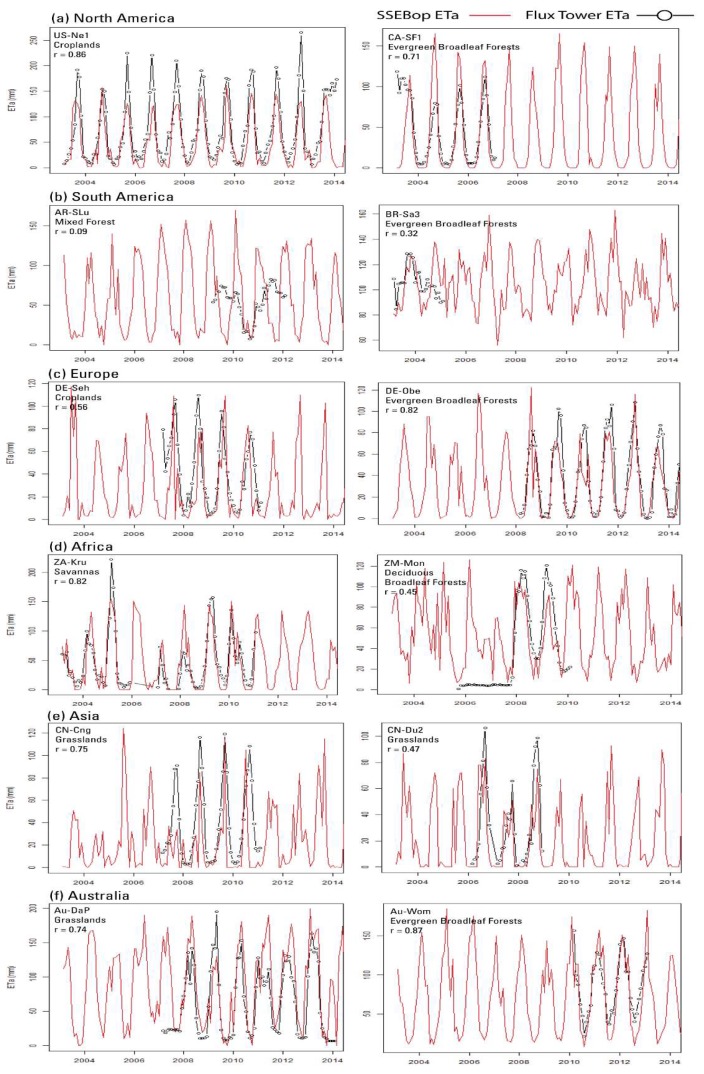
Monthly traces of SSEBop (red) and observed (black) EC flux tower ETa (mm) organized by continent. Two EC towers are shown for each of the six continents. SSEBop is shown from the start (2003) until the most recent available EC date (2014) for visual inspection of year-to-year variability. Land cover type and r values are shown for the flux tower sites.

Despite the concerns with SSEBop ETa biases and EBC error with EC flux tower data, SSEBop ETa captured the temporal variability with a reasonable performance (Figure 4). The observed bias seemed to vary from region to region. SSEBop seemed to under-estimate Europe and Asia, but partly over-estimate Africa and Australia. We can also see the bias appearing to shift between over- and under-estimation in some flux towers such as ZA-Kru (Africa), which casts doubt on the most important strength of the data (consistent bias) for drought monitoring. However, the dry years remained dry (low ETa) and the wet years were wet (high ETa), even with such changes in bias signs, indicating reliability of the SSEBop ETa for drought monitoring.

Temporal traces of both the EC flux tower and SSEBop ETa in Figure 4 show that the seasonality was reasonable for most sites, but highly suspected errors in the EC data are illustrated in AR-SLu (Figure 4b) where the EC data seemed to miss the seasonality, showing a higher ETa in the winter (low SSEBop) than summer. On the other hand, ZM-Mon (Figure 4d) reported almost identical values close to 10 mm for several months in 2006 and 2007, where SSEBop ETa showed the expected seasonality. It is also interesting to note that even when the r was not that strong, such as in the case of CN-Du2 (Figure 4e), SSEBop captured the year-to-year variability when the low EC ETa in 2007 was represented well, indicating the reliability of the SSEBop ETa model as a drought monitoring tool. The same is true in capturing the drought in 2007 for ZA-Kru (Figure 4d). Some of the biases could come as a result of differences in spatial resolution. In looking at the SSEBop ETa for site US-Ne1 (irrigated site), the drought of 2012 in the United States was identified by the SSEBop model as one of the lowest ETa years, but the EC tower data showed the highest summer value, potentially representing a well-irrigated field with high atmospheric demand in a drought year. In this case, both datasets could be right, but representing different spatial scales (Figure 4a).

### 3.3. Evaluation of ETa with Annual Water Budget 

**Figure 5:**
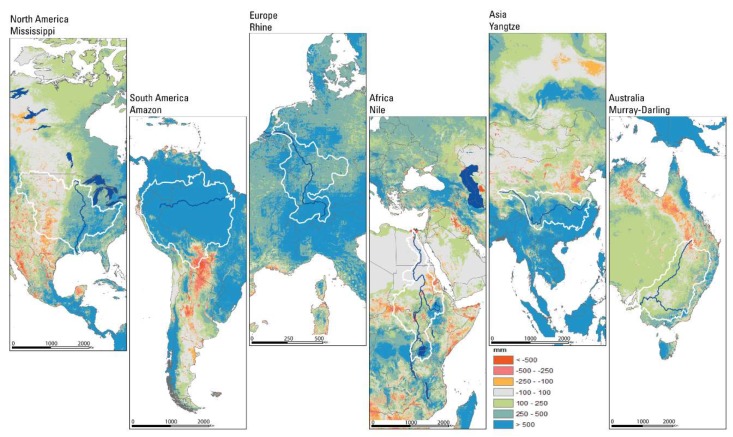
Water balance: annual mean Multi-Source Weighted-Ensemble Precipitation (MSWEP) precipitation data (2006–2015) minus annual mean SSEBop ETa data (2006–2015) for parts of continents including the six major river basins. Orange to red values indicate higher ETa values than precipitation (irrigation and wetlands), while blue-green values show a higher precipitation (PPT) than ETa (rainfed). Water balance is calculated on a per-pixel basis.

Annual water budget evaluations are illustrated in Figure 5 for six continents and six basins. This analysis does not involve runoff and hence only a partial water budget analysis without an attempt to close the budget was performed. Under most circumstances we expect precipitation to be more than ETa; thus, positive differences indicate runoff-producing regions while negative differences imply sinks (more ETa than PPT), where ETa is met by moisture sources other than direct rainfall (e.g., irrigation and shallow groundwater over wetlands and deltas). In Figure 5, the red-orange colored areas represent areas where ETa was more than PPT. While deltas and major irrigation areas showed negative differences, unexpected areas such as the southern part of the Amazon basin and northeast Australia also showed such negative differences, indicating an over-estimation of model bias error, assuming PPT is correct. 

On the other hand, blue-green regions indicate areas where PPT was more than ETa. Such positive (high runoff) areas were in commonly rain-rich areas where major rivers originate. Grey areas indicate areas where the input (PPT) and ETa were about equal (within +/− 100 mm). These regions included The Sahara and Gobi Deserts, where the small amount of precipitation was lost to ETa without generating much surplus runoff. 

Basin-average ETcoeff (ETa/PPT) values provide insight into the basin-scale partitioning of precipitation between ETa and runoff across the six major river basins distributed in diverse hydroclimatic regions (Table 5). Overall, the average ETcoeff ranged between 36% and 82% across the six basins, which is not unusual over diverse hydroclimatic settings [32]. The low (<50%) ETcoeff basins were in rain-rich (Amazon, 48%) or energy-poor, snow-fed (Rhine, 36%) areas while the highest (>80%) ETcoeff value was observed in the rain-poor, high-energy Nile Basin.

**Figure 6:**
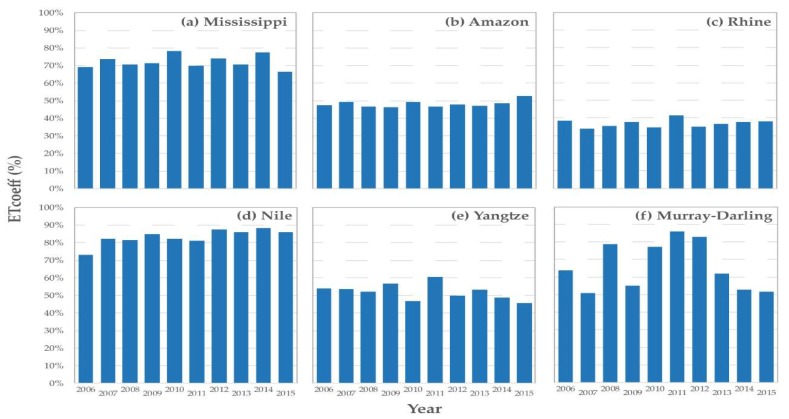
Yearly ETcoeff (ETa/PPT) variability for six major river basins over 10 years (2006–2015).

Most basins showed a relatively minor year-to-year variability in terms of ETcoeff (Figure 6). The Yangtze basin in Asia (Figure 6e) and Amazon basin in South America (Figure 6b) showed a stable 51% and 48% ETcoeff, respectively, over the years, while the Mississippi basin in North America (Figure 6a) showed a stable 72% ETcoeff, which appears to be a reasonable estimate for the region [32]. The clear exception is the Murray-Darling basin in Australia (Figure 6f), which showed a big increase during the relatively wet years of 2010–2012 after a period of drought [33]. The Nile Basin in Africa (Figure 6d) also showed an increase in ETcoeff by about 10% during the 2012–2015 period. The sudden increase in ETcoeff in some years could imply increased rainfall that was converted into ETa, assuming most of the increased PPT over the basin went into ETa compared to runoff. This assertion requires information on runoff data, which is beyond the scope of this study. It is important to note that any error in PPT could lead to erroneous jumps or dips in the ETcoeff as well. However, the overall low ETcoeff (36%) over the Rhine basin in Europe (Figure 6c) may support the under-estimation of SSEBop ETa in relation to the EC flux tower, and suggests the need to apply local or regional bias correction to the SSEBop ETa before using it for water budget studies.

### 3.4. Drought Monitoring Using ET Anomalies

**Figure 7:**
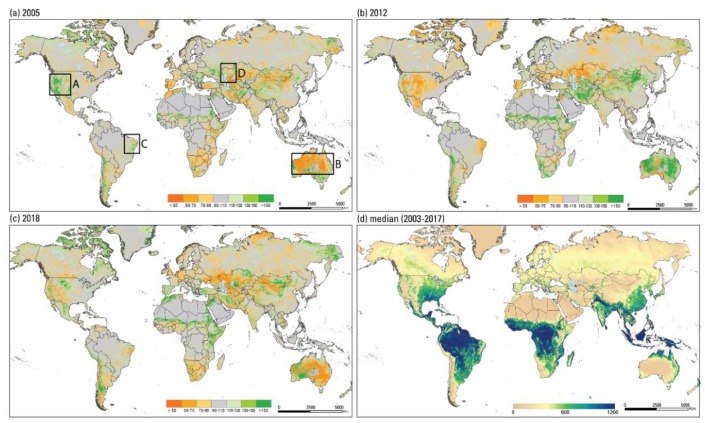
ET anomaly (%) maps for (**a**) 2005, (**b**) 2012, (**c**) 2018, and (**d**) median (2003-2017) actual ET map (mm).

While the absolute magnitude of ETa is critical for water management and hydrologic water budget studies, drought monitoring requires deviations from average patterns in which case any biases in the ETa dataset will drop out during the anomaly calculation. SSEBop ET anomalies (Figure 7a–c) are created as ratios (expressed as percentage) of a particular aggregation period (month, season, or year) to the median of the corresponding aggregation period generated from the 2003–2017 dataset (Figure 7d for annual ETa).

Figure 7a–c show wet and dry year examples in some parts of the world using annual ETa anomalies. Parts of the United States showed a wet trend (green, above normal ETa) in 2005 and dry trend (orange, low ETa) in 2012. The years of 2011/2012 were known drought periods, especially in southcentral United States (Figure 7, box A), caused by below average precipitation [34,35]. Australia had dry conditions in 2005 (Figure 7, box B), which was a period known as the Millennium drought and impacted the basin’s water resources during the first decade of the 21st century [36,37]; wet conditions in 2012 were attributed to the return of precipitation, which ended the drought. Cunha et al. [38] mapped drought severity indices in Brazil’s Amazon basin during 2011–2019 (Figure 7, box C), which corresponds to the extreme drought location shown on the 2012 map in Figure 7b and the moderate drought level identified in 2018 shown in Figure 7c. On the other hand, western Kazakhstan, northeast of the Caspian Sea (Figure 7, box D) shows dryness for all three years (2005, 2012, 2018; Figure 7a–c). Figure 7d shows the median annual ETa distribution from which the yearly (Figure 7a–c) ETa anomalies were created. The USGS FEWS NET posts and consults these products as parts of the convergence of evidence, along with rainfall and vegetation index anomalies, to develop drought monitoring and early warning bulletins in order to avert potential food-insecurity crises. All products (monthly, seasonal, and annual) can be viewed and/or downloaded at http://earlywarning.usgs.gov/fews/.

## 4. Conclusions

The main objectives of this study were to present the methodology used in generating the global SSEBop ETa, along with a limited evaluation using eddy covariance and water budget approaches. The SSEBop ETa product, generated from the Aqua MODIS data stream, is operationally posted on the FEWS NET website (https://earlywarning.usgs.gov/fews/). Data include ETa (mm) at dekadal, monthly, and annual time scales for the globe with the extent shown in Figure 7. ETa anomalies are available for monthly, seasonal, and annual times scales.

Comparison of SSEBop ETa with 12 EC flux tower sites from six major continents showed reasonable performance in capturing the seasonality, but large biases were noted at some sites. Because of the suspected energy balance closure errors, more investigation is required to ascertain the nature and magnitude of the biases. However, the comparison with basin-scale water balance (low ETcoeff) seems to indicate an under-estimation of the SSEBop ETa over the Rhine River basin in central Europe, which would require additional information on runoff and a full water budget analysis.

Despite concerns of bias errors, the SSEBop ETa anomaly can be used for drought monitoring and early warning applications at multiple spatiotemporal scales ranging from 1 km dekadal to basin-scale annual estimates. For water budget studies, the SSEBop data should be considered as a first-order general solution where a one-time local (irrigation region) or regional (basin-scale) calibration procedure could be followed to correct any biases in the SSEBop ETa to produce a region-specific solution before using the absolute magnitudes. 

## Figures and Tables

**Table 1:** Summary of input datasets used in the Operational Simplified Surface Energy Balance Model (SSEBop) model including data sources and the purpose of use. All datasets have a global extent.

	Dataset	Abbreviation	Source	Version	Purpose
1	Land Surface Temperature	LST(or Ts)	MODIS (Aqua)	V6	ETf
2	Maximum Air Temperature	Ta	Daymet/WorldClim	V3/V2	Tc
3	Reference evapotranspiration	ETo	GDAS/IWMI	-	ETa
4	Emissivity	e	MODIS (Aqua)	V6	Ts
5	Normalized Difference Vegetation Index	NDVI	MODIS (Aqua)	V6	Ts, c factor
6	Albedo	a	MODIS	V6	Ts

**Table 2:** SSEBop model parameter description and constraints. LST is the land surface temperature provided by the NASA LP DAAC archive, also denoted by Ts as a variable.

Parameter	Constraints
c factor	NDVI >= 0.7
	Ts > 270 K
	−10 K <= (Ta–Ts) <= 5 K C factor is established under the above 3 conditions
Ts	*albedo correction*
	If a >= 250 & NDVI >= 0 & desert pixel mask
	Then, Ts,a = Ts + 0.1(a -50)
	*Emissivity correction*
	If e > 0.965 & (0.001 < NDVI < 0.25)
	Then, Ts,e = Ts, a(e/0.965)

Note: albedo “a” is scaled by 1000. For example, the LST (320 K) of a pixel with a = 0.30 is adjusted upwards to Ts = 320 + 0.10 (300 − 250) = 325 K. The desert pixel mask was obtained from the Koeppen climate classification (http://koeppen-geiger.vu-wien.ac.at/) [26].

**Table 3:** Monthly comparison of SSEBop ETa and eddy covariance (EC) flux tower ETa for 12 sites globally for 2003–2014. ETa columns are mean values for all months available with matching pairs. RMSEm (%) is the RMSE divided by the mean of flux tower ETa, and RMSEr (%) is the RMSE divided by the range of flux tower ETa. Percent bias is the average difference between the SSEBop ETa and flux tower ETa from matching pairs divided by the mean of EC flux tower ETa; r is the Pearson’s correlation coefficient.

Site	Continent	Land Cover	SSEBop ETa (mm)	Flux Tower ETa (mm)	Range (mm)	Bias (mm)	Bias (%)	RMSE (mm)	RMSEm	RMSEr	r
AU-DaP	Australia	Grasslands	89.7	63.2	188.2	26.5	42%	35.7	56%	19%	0.74
AU-Wom	Australia	Evergreen Broadleaf Forests	77.8	85.4	134.1	−7.6	−9%	13.6	16%	10%	0.87
CA-SF1	North America	Evergreen Needleleaf Forests	53.0	54.7	107.4	−1.7	−3%	17.8	33%	17%	0.71
US-Ne1	North America	Croplands	51.3	76.9	258.1	−25.6	−33%	38.7	50%	15%	0.86
CN-Cng	Asia	Grasslands	22.9	38.0	116.2	−15.1	−40%	13.6	36%	12%	0.75
CN-Du2	Asia	Grasslands	38.4	49.1	102.4	−10.8	−22%	11.3	23%	11%	0.47
DE-Obe	Europe	Evergreen Needleleaf Forests	32.6	40.4	109.2	−7.8	−19%	15.8	39%	14%	0.82
DE-Seh	Europe	Croplands	27.8	41.7	106.3	−13.8	−33%	18.3	44%	17%	0.56
ZA-Kru	Africa	Savannas	44.6	37.7	221.4	7.0	18%	18.3	49%	8%	0.82
ZM-Mon	Africa	Deciduous Broadleaf Forests	52.6	35.1	120.2	17.5	50%	24.7	70%	21%	0.45
AR-SLu	South America	Mixed Forest	59.1	54.6	75.2	4.5	8%	43.7	80%	58%	0.09
BR-Sa3	South America	Evergreen Broadleaf Forests	104.8	106.0	43.8	−1.2	−1%	7.5	7%	17%	0.32

**Table 4:** Energy balance closure (EBC) statistics for each site. #Days column indicates the number of available daily measurements. #Days_low gives the number of days with <70% EBC along with its percentage (%low). #Days_high gives the number of days with >100% EBC along with the corresponding percentage of days (%high).

Site	Continent	#Days	#Days_low	%low	#Days_high	%high
AU-DaP	Australia	2063	12	0.58	1034	50.1
AU-Wom	Australia	992	246	24.8	396	39.9
CA-SF1	North America	1220	316	25.9	479	39.3
US-Ne1	North America	4360	632	14.5	2001	45.9
CN-Cng	Asia	1131	198	17.5	509	45
CN-Du2	Asia	238	11	4.6	120	50.4
DE-Obe	Europe	2451	837	34.2	938	38.3
DE-Seh	Europe	1198	363	30.3	444	37.1
ZA-Kru	Africa	1939	290	14.9	843	43.5
ZM-Mon	Africa	685	17	2.5	322	47
AR-SLu	South America	448	29	6.5	230	51.3
BR-Sa3	South America	1058	18	1.7	523	49.4

**Table 5:** Summary statistics for the six major river basins shown from left to right in Figure 5: basin name, area in km^2^, average annual ETa (mm) and precipitation (mm), and basin-wide ETcoeff (ETa/PPT) (%).

	Basin Name	Area (km^2^)	PPT (mm)	ETa (mm)	ETcoeff (%)
1	Mississippi	2,981,076	812	582	72%
2	Amazon	7,049,948	2339	1120	48%
3	Rhine	185,000	916	328	36%
4	Nile	3,254,555	625	511	82%
5	Yangtze	1,808,589	1119	576	51%
6	Murray-Darling	1,061,469	463	303	65%

## Supplementary Materials

All graphics and data are available online as a USGS data release in Senay et al. [31].
